# Getting underneath the skin: A community engagement event for optimal vitamin D status in an ‘easily overlooked’ group

**DOI:** 10.1111/hex.12978

**Published:** 2019-10-11

**Authors:** Charlotte Lee, Nuttan Tanna, Mitch Blair, Yusuf Yusuf, Hasan Khalief, Monica Lakhanpaul

**Affiliations:** ^1^ UCL Great Ormond Street Institute of Child Health University College London London UK; ^2^ Whittington Health NHS Trust London UK; ^3^ River Island Paediatric and Child Health Academic Centre Imperial College London UK; ^4^ London North West University Healthcare NHS Trust Harrow UK; ^5^ Harrow Association Somali Voluntary Organisation Harrow UK; ^6^ Present address: Nuffield Department of Primary Care Health Sciences University of Oxford Oxford UK

**Keywords:** barriers, deficiency, knowledge, minority, patient and public involvement and engagement, PPIE, promoters, vitamin D

## Abstract

**Background:**

Patient and public involvement and engagement (PPIE) is recognized as important for improved quality in health service provision and research. Vitamin D is one area where PPIE has potential to benefit public health initiatives, particularly for women and children with increased skin pigmentation (ie at high risk of deficiency) who are easily overlooked.

**Objective:**

We report findings from a community PPIE event that explored the knowledge, barriers and promoters for optimal vitamin D status amongst an exemplar high‐risk and easily overlooked population group.

**Methods:**

Two researchers and one PPIE lead facilitated a single group discussion with twenty members of the Somali community from across west London. All attendees were women of reproductive age, or knew a mother and child that could benefit from a targeted initiative. The discussion was recorded, transcribed verbatim, organized and coded using NVivo 12 Pro to identify emergent themes underpinned by the Health Behaviour Model.

**Results:**

Attendees thought community safety and competing demands of technology and education impacted on sun exposure and lifestyle activity. Language barriers impacted on access to health care. Attendees also felt the mother figure was ‘the most important’ influencer of both child and wider community health.

**Discussion:**

Although further discourse is needed, this event emphasizes that it is important that the public voice is heard in informing, designing and evaluating appropriate public health interventions amongst specific ethnic groups. Insights from this Somali population have suggested benefit from using verbal health messages that are specifically targeted at mothers, compared with the general population.

## 1 INTRODUCTION

Patient and public involvement and engagement (PPIE) is gaining international foothold. Research activities carried out ‘with’ or ‘by’ the public range from design, implementation, evaluation and dissemination.^1^ Patient and public involvement and engagement provides a platform to hear the patient and public's voice, particularly those of minority, seldom heard or easily overlooked groups. Easily overlooked groups with a lived experience of a condition and/or disease often experience different barriers to service access, are under‐researched and are vulnerable to health inequalities. The Department of Health (DoH^2^) and the National Institute of Health Research (NIHR^3^) recognize PPIE as essential for relevant and patient‐focused health care. The PPIE evidence base has grown over the past decade, and evidence is now turning to its impact.^4^, ^5^

For vitamin D deficiency, the patient voice deserves notice because it has now reached epidemic proportions, with over 1 billion people affected worldwide.^6^ Natural dietary sources of the vitamin are limited, and the biggest source of the prohormone 25(OH)D is sun exposure. Pregnant women and their breastfed neonates are at high risk due to the bi‐directional influence of feto‐maternal vitamin D stores.^7^ Deficient (<25 nmol L^−1^) or insufficient (<75 nmol L^−1^) concentrations of 25(OH)D 8 in pregnant women have been associated with lower neonatal birth weight, length and head circumference.^9^, ^10^, ^11^ Hypo‐calcaemic seizures, rickets and cardiomyopathy have also been reported in deficient children under 5 years.^12^ Lastly, randomized controlled trials report the beneficial effects of supplementation on mental health 13 and risk for some non‐communicable diseases including asthma,^14^ type 2 diabetes 15 and cardiovascular disease.^16^

People living in Northern latitudes like the United Kingdom (UK) are also at high risk of vitamin D deficiency. This is because the short duration of ultraviolet B (UVB) light in winter months is insufficient for cutaneous synthesis to occur.^17^ Furthermore, people with increased skin pigmentation, that is type V and above, are considered high‐risk groups. The pigment in their skin, melanin, acts as a natural sunblock.^18^ Conservative clothing worn for cultural or religious reasons may also block light penetration, as does sunscreen. As such, pregnant women and children from South Asian, African, Caribbean and Middle Eastern ethnic minority groups living in the UK are considered at even higher risk, than compared with the general population.^19^, ^20^, ^21^ Despite some specific mechanisms remaining uncertain in relation to hypovitaminosis and the above health outcomes in specific ethnic minority groups, bone and heart health in infants is a valid justification for effective population health intervention. Hence, there is an opportunity to better involve and engage high‐risk groups who can also be easily overlooked in how to improve their vitamin D‐dependent health status.

Both the Scientific Advisory Committee on Nutrition (SACN^22^) and NICE^8^ recommend information and advice provision on supplement benefits for pregnant and breastfeeding women and children under 4 years.^23^ The DoH states that people with increased skin pigmentation should supplement vitamin D. The UK's Healthy Start Scheme has attempted to optimize the nutritional status of pregnant women and their children via food vouchers and supplement coupons. However, uptake has been described as poor, ranging between 3% and 10%.^24^, ^25^, ^26^ The limited understanding, accessibility and acceptability of the scheme by health‐care professionals and families alike may explain these findings.^27^ Furthermore, there is scarce literature surrounding the patient's voice in vitamin D deficiency management, especially among pregnant women of ethnic minority groups. This knowledge gap has hampered the implementation and effectiveness of preventative initiatives.^28^

A number of questions remain to be answered.^29^ What is the current knowledge of vitamin D? What barriers and promoters exist to optimal vitamin D status? Lastly, how may barriers be overcome as proposed by high‐risk and easily overlooked groups themselves? Without first answering these questions, public health initiatives will remain untargeted and ineffective at a cost to growing health inequalities and public expenditure budget.

### 1.1 Aim

We aim to report the current knowledge, and perceived barriers and promoters, for optimal vitamin D status in women and children under 5 years of Somali origin shared during a community PPIE event. This population is an exemplar easily overlooked group at high risk of vitamin D deficiency^18^ because infant vitamin D stores are largely dependent on maternal stores and because of their increased skin pigmentation (type VI). Both women of reproductive age and infants of deficient mothers are at high risk of deficiency and therefore require significantly more sun exposure or dietary supplementation than fairer skinned populations (types I‐IV) for similar vitamin D synthesis. We draw out themes underpinned by constructs of the Health Belief Model (HBM).^30^ Opinions and experiences shared during this PPIE event will prioritize a research question to develop a targeted and relevant public health initiative for optimal vitamin D status in high‐risk groups.

## 2 METHODS

### 2.1 Attendees

The UK has the largest Somali community in Europe, and the Office for National Statistics (ONS) estimates that 98 000 Somali‐born immigrants were resident in the UK in 2016.^31^ More than half—around 66% (n=65 333)—live in London, followed by smaller communities across Birmingham (n=7765), Bristol (n=4947) and Manchester (n=3645). However, estimates vary given the mobility of this population, with many returning to Somalia for extended periods during the summer. Previous qualitative research specifically with the UK Somali population has reported poor awareness as a barrier to hepatitis B testing,^32^ the influence of cultural beliefs and stigma on help‐seeking behaviour,^33^ and the process of co‐producing knowledge about autism.^34^ For this PPIE event, we invited members of the Somali community from across the London boroughs of Ealing, Brent and Harrow between November 2017 and January 2018.

### 2.2 Setting and design

A single PPIE event was run based on available resources at the Harrow Association of Somali Voluntary Organisation (HASVO; www.hasvo.org) centre—a not‐for‐profit organization that aims to strengthen links between ethnic minorities in Harrow, west London. Harrow Association of Somali Voluntary Organisation offers free and confidential advice and information, activities for young children and families, and support around education and employment. We chose HASVO as the setting for the PPIE event because it is a non‐academic, open plan meeting space that is easily accessible by the community. An open group discussion is a suitable method for exploring the opinions of easily overlooked groups that typically would not respond to a postal survey and/or may be intimidated by a conventional interview situation,^35^ and HASVO has experience in running workshops of this nature.

### 2.3 Stages and level of involvement and engagement

#### 2.3.1 Involvement

Two PPIE leads (YY, HK) of the HASVO centre were actively involved in the design, delivery, evaluation and dissemination of the PPIE event. First, the event's design was discussed during a pre‐event, face‐to‐face meeting with both PPIE leads. The PPIE leads confirmed what refreshments would be culturally suitable to provide and that a mixed‐gender session would be acceptable to attendees. Recognizing that the attendees were potential child caregivers, YY and HK advised that the event be run after the morning school run but before noon when many young children return from nursery and/or school. The PPIE leads also advised against running the event on a Friday when many male attendees would attend Friday prayer at their local mosque. Finally, they highlighted a YouTube video designed by Norwegian health professionals on vitamin D in the Somali language,^36^ which we could consider using for information provision.

Next, the PPIE leads used word of mouth to conveniently invite potential attendees using a snowball technique, from across a range of characteristics (ie sex, age, marital status). Four categories reflected potential child caregivers: (a) women of reproductive age, pregnant women or mothers, (b) fathers, (c) grandparents and (d) business, religious and media leads. There were no specific exclusion or inclusion criteria since we aimed the event to be accessible and open to anyone willing to express their opinions. All attendees identified themselves as Somali and self‐reported a basic level of English proficiency. The targeted and final number of attendees (N=20) was deemed by the authors as acceptable to reach a balance between information quantity and quality, that is capacity to analyse.^36^

#### 2.3.2 Engagement

CL and NT developed a topic guide (Appendix S1) to help engage attendees and cover issues without leading opinions, but remain sensitive to unsolicited themes. The topic guide was underpinned by components of the HBM.^30^ Briefly, the HBM attempts to explain and predict health‐related behaviours across the following four constructs: (a) perceived susceptibility (ie known risk of deficiency), (b) perceived severity (ie known consequences/health outcomes of deficiency), (c) perceived barriers (ie sufficient sun exposure, supplementation and health information access) and (d) perceived benefits (defined here as promoters for optimal vitamin status, that is sufficient sun exposure, supplementation and health information access). In addition, we explored recommendations for future health initiatives and interventions.

Edits from MB and ML were incorporated into the topic guide, followed by a pilot with an external researcher. Both PPIE leads reviewed the final topic guide with no further comments and helped co‐produce the Demographic Questionnaire for cultural appropriateness. This led the PPIE leads to suggest including khat usage, which is a leafy green plant containing a Class C illegal stimulant that is often chewed as a cultural tradition.^37^ Two presentation methods facilitated group discussion. First, Microsoft PowerPoint slides with culturally and ethnically appropriate cartoon pictures promoted discussions pertaining to the attendee's perceived susceptibility and severity of vitamin D deficiency. Second, the topic guide prompted open discourse around the perceived barriers and promoters to different sources of vitamin D and health information access. The discussion ended with recommendations on targeted health messages.

### 2.4 Procedure and information capture

All attendees were invited to complete the Demographic Questionnaire. The discussion was recorded using two digital recorders. All attendees were reminded that their personal identifiable information would remain confidential and that they could leave at any time without having to give a reason. Two members of the research team (CL, NT) and one PPIE lead co‐ran the discussion in English, with Somali translation available via both PPIE leads. The discussion lasted approximately two hours. At the end of the event, attendees were shown the recommended YouTube video^38^ and were also provided with the Public Health England vitamin D leaflet.^39^ In line with the INVOLVE good practice guidelines,^1^ all attendees were provided with a store voucher as financial reimbursement to cover their attendance time and contribution.

### 2.5 Reporting

An unbiased professional transcribed the recorded discussion verbatim. Where audio was compromised, the second recorder was played and/or confirmed by a member of the research team (NT). To ensure transcription accuracy, CL checked the transcript against the recording. A thematic analysis method^40^ was used to draw out themes and sub‐themes underpinned by constructs of the HBM. Each member of the research team analysed transcripts separately. As CL and NT facilitated the PPIE event, peer debriefing with two further researchers (MB, ML) was used to vet the analysis. For improved inter‐rater reliability, all four researchers cross‐referenced their themes. Next, themes were validated by an external reviewer (TM). From this, a single unified framework was reached and ratified by both the PPIE leads. All analyses were conducted in NVivo 12 Pro^41^ to facilitate information organization and audit. This event is reported in accordance with the Guidance for Reporting Involvement of Patients and the Public long form [GRIPP2‐LF].^42^

## 3 OUTCOMES

### 3.1 Attendee characteristics

Demographic characteristics are available in Table 1.

**Table 1:** Demographic characteristics (N = 20)

Demographic characteristics (N=20)	n (%)	N (100%)
Gender		
Male	9 (45%)	20 (100%)
Female	11 (55%)	
Age		
18‐24	1 (5%)	19 (95%)
25‐33	4 (20%)	
34‐49	8 (40%)	
50‐64	4 (25%)	
65+	2 (10%)	
Marital status		
Single	5 (25%)	20 (100%)
Married	12 (60%)	
Divorced	2 (10%)	
Widow/widower	1 (5%)	
Educational level		18 (80%)
GCSE & A levels/equivalent	4 (20%)	
Diploma	3 (15%)	
Undergraduate	3 (15%)	
Postgraduate	1 (5%)	
No/other	7 (35%)	
Place of birth		20 (100%)
Somalia	18 (90%)	
UK	0 (0%)	
Other	2 (10%)	
No. of children		
0	1 (5%)	17 (85%)
1	0 (0%)	
2	2 (10%)	
3	1 (5%)	
4+	13 (65%)	
Children's place of birth		
Somalia	4 (20%)	17 (85%)
UK	9 (45%)	
Both Somalia & UK	2 (10%)	
Other	2 (10%)	
Alcohol consumption		
Yes, regularly	0 (0%)	20 (100%)
Yes, occasionally	0 (0%)	
No, not anymore	1 (5%)	
No, never	19 (95%)	
Tobacco consumption		20 (100%)
Yes, regularly	2 (10%)	
Yes, occasionally	0 (0%)	
No, not anymore	5 (25%)	
No, never	13 (65%)	
Khat consumption		20 (100%)
Yes, regularly	1 (5%)	
Yes, occasionally	0 (0%)	
No, not anymore	4 (20%)	
No, never	15 (75%)	

### 3.2 Emergent themes

The analysis yielded three main constructs (knowledge and attitudes, barriers and promoters) and four themes (nutrition, sun exposure, supplementation and health information access; Table 2).

**Table 2:** Identified constructs and themes

	Knowledge & attitudes	Barriers	Promoters
Nutrition	Nutritional sources of vitamin D Potential positive attitudes to food fortification	Generational differences in opinions to healthy nutrition	Healthy Start Scheme for food coupons
Sun exposure	Lack of sunlight and darker skin pigmentation as risk factors for vitamin D deficiency Health consequences of vitamin D deficiency	Weather Erroneous or mixed health messages Community safety and competing demands of technology and education as a barrier for lifestyle activity	Outdoor play facilities for older children Garden access Expectations of exercise and sun exposure in Somalia
Supplements	Impact of supplements on health Lack of knowledge that supplementation may be self‐managed	Tablets as a form of administration Stigma and financial constraints associated with elf‐supplementation	Healthy Start Scheme for vitamin D supplements
Health information access	GP as the main source of health information Outreach workers and midwives as additional sources of health information	Language barriers Perception of pharmacies as dispensers of medication, rather than being able to offer advice	Verbal communication via media School engagement via nurse‐led training to mothers and the child's educational curriculum Focus groups using community champions Mother figure as a key influencer within the community

#### 3.2.1 Construct 1: knowledge & attitudes towards nutrition, sun exposure, supplementation and health information access

Attendees understood that ‘fruit and veg[etables]’ were important for optimal nutrition. However, some erroneously believed that consuming ‘a lot of fish’ and ‘milk’ were adequate sources of vitamin D, based on advice from their general practitioner (GP). Cow's milk is not fortified with vitamin D in the UK. However, their belief in and continued consumption of milk are suggestive of positive views towards fortification, although this was not probed.

Attendees understood that they are at high risk of deficiency owing to their skin pigmentation—‘you cannot absorb more light’. They also understood that increased sun exposure would improve their vitamin D status ‘because we don't get enough sun’. Furthermore, attendees were aware that ‘broken bones’ and ‘fatigue’ are health outcomes of vitamin D deficiency in adults, but not children.

Almost half (9/20) of the attendees were taking vitamin D supplements; seven did so based on their GP’s prescription. A central issue involved self‐supplementation. Most did not understand that supplementation would need to be self‐managed when ‘the doctor stopped’ the vitamin D prescription. In fact, attendees thought that they did not ‘need it anymore’. This may be partially explained by medicalized views towards supplementation, that is use as a ‘cure’ rather than as a ‘preventative’ approach. These attendees viewed vitamin D deficiency as needing prescribed treatment as opposed to long‐term supplementation. Five attendees agreed that the GP was their main source of health information. Outreach workers and expert teams like the researchers were known sources of health information (Appendix S2).

#### 3.2.2 Construct 2. Barriers towards nutrition, sun exposure, supplementation and health information access

Three attendees agreed that the cartoon depicting a seemingly healthy child was ‘too skinny’, that overweight children were ‘nice to cuddle’ and that ‘the chubbier the child is the healthier the child is’, reflecting misunderstandings around optimal child health. However, two younger attendees felt pressured to ‘feed him, feed him’ by older relatives, suggesting potential generational differences with regard to appropriate nutritional intake.

On why they do not expose their skin in the sun, two attendees agreed that they ‘don't get the opportunity’ to go in the sun. Specifically, outdoor play and socializing in Somalia were viewed as more feasible due to improved safety and lack of transport facilities, than compared with the UK—‘a lot of these kids are ending up in gangs and dealing drugs’. Three attendees thought technology (eg ‘he just wants to sit down and play with his tablet’), and education and tuition (eg ‘they come back [from school] and do homework, and TV a little bit’) influenced the amount of outdoor physical activity for children in the UK. Attendees also cited reminders by their children's school to apply sunscreen during the British summer. One attendee recalled a health professional's advice that ‘the sun in this country is not very healthy’. There were mixed reported barriers across the eleven attendees who were not taking supplements, ranging from ‘I'm not of the belief in supplements’ from one grandparent possibly due to the stigma related to medicines among older generations, ‘I just don't like tablets’ from one middle‐aged attendee, and ‘I don't have access to it’ from a younger attendee. Another attendee commented that they ‘did not want to search for it’ in pharmacies or that they ‘wouldn't get one specifically for vitamin D’. Three mothers found it ‘almost impossible’ for their children to swallow supplements.

Pharmacies were not favoured as health information access points, suggestive of stigma or language barriers around information access (eg ‘we tend to feel shy’) or even financial barriers associated with self‐supplementation. A GP prescription may be free and discretely available, whereas a pharmacy would involve an ‘over the counter’ purchase. Others cited pharmacists as typically dispensing medication, as opposed to offering advice and offering medication (Appendix S3).

#### 3.2.3 Construct 3. Promoters of nutrition, sun exposure, supplementation and health information access

There were mixed opinions on whether women removed their hijabs for sun exposure—‘I don't do that’ vs ‘they do actually go in their back garden during the summertime and they do expose more of their skin to the sun’. There were preferences for taking children ‘to the park’ and outdoor ‘playground areas’ for sun exposure.

Only one attendee had heard of and used the Healthy Start Scheme for food coupons and supplements, despite all attendees knowing a potentially eligible child. That same attendee expressed interest in supplement injections seeing it as more convenient in the long‐term because s/he ‘always forgets’ supplement tablets.

Attendees held strong views about delivering health messages in the community. They expressed a need for a two‐pronged approach: ‘there's the initial message and then there's the actual conversation’. Prior work had been conducted within the west London Somali community around diabetes using community awareness sessions. To date, there is no impact data available on this work. However, attendees felt a similar model would ‘absolutely’ work for vitamin D. In particular, attendees wanted ‘somewhere they can feel safe to come together as a community’ to receive targeted health messages ‘in sessions… like what we're doing now’. Three others suggested initiatives should ‘use media, for example WhatsApp or the Somali [TV] channel’ for public health messages. This may be partially explained by Somalis being a ‘word of mouth’ community and ‘not very confident in communicating with the outside world’. Attendees suggested that schools and nurses could be ‘a bit more engaged in terms of training parents’ around child health outcomes and that health awareness could be taught within the ‘educational curriculum’ like ‘in Africa’. There was unanimous agreement that ‘the source of information within the Somali community is the mother… if the mother is educated enough and knowledgeable enough… then the community is educated’. Others agreed, ‘there is a need to educate mum’ and that the mother is ‘the most important person’ in optimizing child and community health (Appendix S4).

## 4 DISCUSSION

This PPIE event provided information on the current knowledge, and perceived barriers and promoters towards optimal vitamin D status, and offered suggestions for public health initiatives. Several key issues were identified: first, poor‐ or misunderstandings around optimal child health; second, medicalized views towards supplementation; third, concerns around drug and gang crime, and competing demands of technology and education as a barrier for lifestyle activity; fourth, difficulties in remembering to take and swallow tablets for both adults and children, and possible financial barriers on self‐supplementation; fifth, language barriers; and finally, mothers as an influencer of child and community health. Underpinned by the HMB^30^ and guided by the GRIPP2‐LF,^41^ this event may serve as an example for researchers, clinicians and public contributors on how to incorporate and report PPIE events for improved quality, consistency and transparency in patient‐centred research^5^

Research on vitamin D with other high‐risk and easily overlooked groups including South Asians across east London^29^ and Manchester^43^ has reported similar outcomes. Knowledge may be inconsistent given the invalid and/or inconsistent messages available on the Internet (eg milk as a source of vitamin D), conflicting public health campaigns (eg CANCERactive Safe Sun campaigns), and limited access to evidence‐based advice from health professionals. Other work on asthma in South Asians has shown that health professionals often focus on medicinal rather than behavioural approaches to self‐management^44^, ^45^ and that culturally appropriate educational interventions are effective for long‐term improved health outcomes.^46^ More recent evidence suggests that it is feasible to deliver a community‐based intervention to optimize vitamin D knowledge and medication adherence in South Asians^47^ and that it is important to develop a culturally appropriate educational package.^48^

In addition, although Harrow crime rates are below the national average^49^ attendees felt community safety impacted on outdoor activity and sun exposure, with concern that their children would interact with gangs and either use or deal drugs. These barriers may be an even greater cause for concern in other UK regions. Cultural influences (ie medicalized views towards supplementation), minority status and potentially associated poor English proficiency may further exacerbate barriers for access to good quality health information.

We found 19/20 attendees had not heard of the Healthy Start Scheme despite knowing a potentially eligible child under 5 years. Previous studies have reported concerns regarding the low uptake of the scheme's vitamin supplements.^27^ Explanations include the restrictive nature of eligibility criteria and complex administrative processes.^25^ Universally implementing the Healthy Start Scheme may be more effective in overcoming stigmatic barriers to self‐supplementation^27^ although this approach may have only minimal (17%) improved uptake,^50^ especially given that our group reported difficulties swallowing tablets. Universal fortification represents an alternative population‐level approach. Our event noted tentative support for fortification, which merits further in‐depth exploration and consideration of alternative methods of delivery (ie mouth spray, skin delivery systems). National fortification offers a cost‐effective preventative strategy for reduced health burden but remains unpalatable in the face of population resistance with freedom of choice, coercion, safety and trust as commonly highlighted concerns.^51^

Group discussions are useful in obtaining detailed information about personal and/or group attitudes and experiences. Patient and public involvement and engagement leads co‐designed and facilitated the event in Somali, which enhanced cultural appropriateness and potentially yielded more detailed information. Interviews were digitally recorded and professionally transcribed to reduce researcher bias. The external researcher enhanced validity through consensus decision making on developing the framework of themes. Nonetheless, our work has some limitations. As with all group discussions, dominant opinions may overshadow the response of others.^35^ Second, the PPIE leads and some attendees had previous experience with some members of the research team^52^ and hence may have had greater and unrepresentative understanding of vitamin D compared with the target population. If so, there would be greater cause for concern about community knowledge than compared with our findings. We also cannot rule out reporter bias. Third, although the facilitators were trained against asking leading questions and giving affirmative responses, which may influence subsequent responses, researcher bias cannot be entirely excluded. Fourth, variable English proficiency may have led to communication difficulties for some attendees, especially where conversation was fast paced or complex in detail. Although attendees were aware of a facilitator's ability to translate into Somali if needed, no attendees asked for translation. Fifth, an additional event could have yielded advice from patients and the public on the topic guide to ensure it was relevant, understandable and focused. We did not offer this additional event given available resources. Lastly, we did not find ethnic‐specific issues, which may reflect our topic guide, design of the event or limited probing of some statements given the large group session and time constraints. Future PPIE events may consider debriefs with attendees. However, our identified themes point towards possible exploration in future, with more in‐depth qualitative research.

## 5 CONCLUSION

Patient and public involvement and engagement represents an opportunity for researchers to ensure patient‐focused health research and service provision. For vitamin D deficiency, it is important that coherent and targeted public health initiatives are developed for high‐risk and easily overlooked groups, as supported by the DoH, SACN and NICE. With regard to current schemes, clearly one message does not fit all. Our PPIE event serves as a valued opportunity to co‐develop—with the community—appropriately tailored and designed public health interventions. Additional published discourse with communities and health‐care professionals is essential to further explore the underlying mechanisms by which barriers and promoters impact on vitamin D status for the UK's most easily overlooked groups.

## CONFLICT OF INTEREST

All authors declare that there are no competing interests.

## AUTHORS' CONTRIBUTIONS

All authors conceived the study and elaborated the design. YY and HK helped coordinate and manage the event. CL, NT and MB analysed the information, and all authors contributed to its interpretation. CL wrote the manuscript. All authors critically reviewed the final manuscript. ML is the guarantor.

## ETHICAL APPROVAL

This work was undertaken in the context of patient and public involvement and engagement (PPIE) and did not require formal ethical or Health Research Authority approvals.^41^ This work served as a pilot prioritization exercise undertaken to inform the River Island Paediatric Academic Unit's ‘Vitamin D Life Course’ study with London North West Healthcare University National Health Service (NHS) Trust as sponsor and under Research & Development (R&D) approval for service development and evaluation, reference R&D SE16/013.

## CONSENT TO PUBLISH

All attendees consented to sharing their opinion, for session recording and for the facilitators to use quotes from the discussion for the purpose of this article. Attendees understood that they could leave the event at any time and that they would not be personally identified in any report.

## Data Availability

The information that supports the outcomes of this work is available from the corresponding author upon reasonable request.
