# Supplementary material for: Getting underneath the skin: A community engagement event for optimal vitamin D status in an ‘easily overlooked’ group
Source: Health Expect. 2019 Oct 11;22(6):1322–30. doi: 10.1111/hex.12978 (PMC6882264; doi:10.1111/hex.12978)
Supplement: Supplementary file 1 [file HEX-22-1322-s001.docx]

# LIST OF SUPPLEMENTARY MATERIAL

## **Appendix S1. Topic guide for semi-structured group discussion**

| **Concept** | **Question** | **Actions and Example Prompts** |
| --- | --- | --- |
| **Ice-breaker** | Let’s start with everyone telling us their name, whereabouts they were born and, if appropriate, how long have you been here in the UK.  I’ll [facilitator] go first… |  |
| **Introduction / perceived severity** | What does a ‘healthy child’ mean to you? | *Action: show PowerPoint examples of different infants and children.*  How does a healthy child look and behave? E.g. is s/he tall, short, slim, chubby, active, quiet, chatty? |
| **Perceived susceptibility** | What type of foods do you think are good or bad for children, and how did you get these information or beliefs around good or bad? | *Action: show PowerPoint examples of fish, eggs, crisps, ice cream.*  *NB: these are low sources of vitamin D.* |
| **Story - personal experience** | I’m going to tell you a story. As I go along you can add your views for the child of that age.  Basra is 5 years old. Since she started walking Basra’s mother has noticed she walks differently to other children at school (e.g. curved legs - [indicate with gesture]). Basra now always seems tired. Her mother has been feeding her sugary foods to increase her energy but Basra already has problems with her teeth.  Any thoughts or comments? Is it something you have seen in real life? If your child experienced something similar what would you do? | House without garden, not allowed to play outside (safety worries), dental caries. |
| **Perceived severity** | We know that some children have problems with their teeth and may later develop rickets, obesity, diabetes and depression.  Do you think these are big problems in your community? If yes, do you think there is something we can do to help in you and the children in your community to prevent these health problems? | In what ways do you think being in the sun matters to health?  In what ways do you think food matters to health?  Where do you go / who do you contact for information about your child’s health? |
| **Perceived benefits** | Can everyone think of a child that they know? Are they between 0-5 years? Can you describe when and how the child is/was exposed to sunlight?  I’ll [facilitator] go first. I’m thinking of my niece…. | When do they go in the sunlight?  How long do they remain in the sunlight for?  What do they do when in the sunlight?  Are they usually covered up with clothes when outside / is sunscreen put on them?  Does how long / when / what they do in the sunlight depend on age e.g. pre-nursery / pre-school?  What you think may influence the parent’s choices or decisions around sun exposure?  Further probe:  Availability of sunlight, own culture, surrounding culture, family, religion, length of stay in the UK, education/ schooling etc. |
| **Perceived benefits and/or barriers**  **Perceived benefits and/or barriers**  **Perceived benefits and/or barriers** | Do you think that being Somali means that you think differently about vitamin D to someone who is not from this background? If so, how? | Are there any sociocultural factors?  English proficiency?  Knowledge of British healthcare system?  Other priorities e.g. housing?  Lifestyle: exercise, diet, stress, smoking (khat), alcohol? |
|  | Where do you think people in your community go for more information?  *NB: this may come out earlier. If yes, do not repeat. If no, then can be asked here.* | Where do you get your information from that makes you do what they do regarding children’s health?  Do you think being born in the UK or Somalia/ length of stay/ male or female/ fluency in English makes a difference to your health? |
|  | What about the role of online and social media, and community leaders? | Do you use WhatsApp, Facebook, community centres? |
| **Recommendations for future health interventions** | Is there anything that doctors/ nurses/ other healthcare professionals can do to help make your children healthier? | How could we better educate people about child health?  What could other professional (e.g. health visitors, midwives, community pharmacists) do to help to make people listen to their advice?  If they were to try to get the information across what should they do or say?  Do you think it is easy for Somali mothers who have little English literacy to benefit from NHS services? Why or why not? What may help: Women’s group talks, radio messaging, YouTube videos, leaflets, Somali newsletters, WhatsApp groups? |
|  | You may have heard of some guidelines for young children and their vitamin D. If yes, have you been taking the vitamins and using the vouchers? If not, what has stopped you from using the scheme? | E.g., PHE leaflet, Healthy Start. |
| **Close** | Lastly, are there any final thoughts or experiences that you would like to share? |  |
