# Supplementary material for: Getting underneath the skin: A community engagement event for optimal vitamin D status in an ‘easily overlooked’ group
Source: Health Expect. 2019 Oct 11;22(6):1322–30. doi: 10.1111/hex.12978 (PMC6882264; doi:10.1111/hex.12978)
Supplement: Supplementary file 2 [file HEX-22-1322-s002.doc]

# LIST OF SUPPLEMENTARY MATERIAL

## Appendix S2. Construct 1, Knowledge & Attitudes

| **Themes** | **Quotes** | **Attendee** |
| --- | --- | --- |
| **Nutrition** | “*Meat is not bad. But eating too much red meat is not good. Maybe fish and chicken is better*” | Male, middle-aged |
| **Sun Exposure** | “*Many with darker skin have to wait without sun cream for 20 minutes. That’s what I’ve been told. But in this country I don’t get that and I don’t get that opportunity to even do it*” | Female, young |
|  | “*They can go in the garden and maybe just take the* [gestures to remove hijab]” | Male, middle-aged |
|  | “*They do actually go in their garden during the summer and they do exposure more of their skin to the sun*” | Male, middle-aged |
|  | “*We actually get our source of sun when we go back home*” | Female, middle-aged |
| **Supplements** | “*She’s trying to give vitamin D supplements to all of her children, but… her children doesn’t want to take the vitamin D supplements… but she finds it almost impossible*” | Interpreter on behalf of a female, middle-aged |
|  | “*I’ve been prescribed the tablets and I always forget them. I put them in my bag, that doesn’t work, in the cupboard, next to my bed. Nothing works. I am always forgetting*” | Female, young |
