# Supplementary material for: Getting underneath the skin: A community engagement event for optimal vitamin D status in an ‘easily overlooked’ group
Source: Health Expect. 2019 Oct 11;22(6):1322–30. doi: 10.1111/hex.12978 (PMC6882264; doi:10.1111/hex.12978)
Supplement: Supplementary file 3 [file HEX-22-1322-s003.doc]

# LIST OF SUPPLEMENTARY MATERIAL

## Appendix S3. Construct 2, Barriers

| **Themes** | **Quotes** | **Attendee** |
| --- | --- | --- |
| **Sun Exposure** | “*Back home that generation used to… exercise as part of their daily routine. But right now when you… tell them you need to go to the gym to exercise, where’s that logic? We used to walk and be active for a reason and the thing is now… kids are taken by buses to go to school where that wasn’t happening before*” | Male, middle-aged |
|  | “*In the past… you can leave the kids… over the years… a lot of the families have realised that… a lot of these kids are ending up in gangs and dealing drugs and things like that, so now they’ve become a lot more protective of them. So, what it’s done is limit the physical activities that the kids will get into, because now the mother feels that she needs to be present wherever that child is and you can only be there so much”* | Male, middle-aged |
|  | “*Kids born now… they are getting influenced with the technology. So now my little nephew who is a year and a half he knows how to go on YouTube and how to play. So, he just wants to sit down and play with his tablet*” | Female, middle-aged |
|  | “*Now there is a push in the community to excel in education… the parents now are concentrating on ‘now I need to take my child to tuition’ so the amount of time that is left in the evening for a child to do any football is not there*” | Male, middle-aged |
| **Supplements** | “*I’ve been prescribed the tablets and I always forget them. I put them in my bag, that doesn’t work. In the cupboard, next to my bed. Nothing works. I am always forgetting*” | Female, young |
|  | “*She’s trying to give vitamin D supplements to all of her children. But one thing that she has noticed is that one of her children doesn’t want to take the vitamin D supplements. She finds it almost impossible to get him to take any supplements of vitamin D*” | Interpreter, on behalf of young female |
|  | “*I don’t take it because I just don’t like tablets*” | Female, young |
|  | “*It’s not the fact that I don’t like tablets, but I just add water and let it go down. I don’t have access to it. Literally, alright I’m lazy in that kind of thing, I don’t want to search for it, do you know what I mean?”* | Male, young |
| **Health Information Access** | “*Language is a barrier amongst Somali’s actually. We tend to feel shy and we are not very confident in communicating with the outside world. We remain within our own comfort zones, that’s where we get out information from*” | Male, middle aged |
|  | “*We, culturally we tend to see the pharmacies as dispensing the medicine so we wouldn’t be there to ask for medication*” | Male, middle aged |
|  | “*I wouldn’t go and ask the pharmacy first. I would more for the GP*” | Female, young |
