# Supplementary material for: Getting underneath the skin: A community engagement event for optimal vitamin D status in an ‘easily overlooked’ group
Source: Health Expect. 2019 Oct 11;22(6):1322–30. doi: 10.1111/hex.12978 (PMC6882264; doi:10.1111/hex.12978)
Supplement: Supplementary file 4 [file HEX-22-1322-s004.doc]

# LIST OF SUPPLEMENTARY MATERIAL

## Appendix S4. Construct 3, Promoters

| **Sub-Theme** | **Quotes** | **Attendee** |
| --- | --- | --- |
| **Media** | “*There’s the initial message and then there’s the actual conversation about. I think giving the message is quite easy. You can have it there. You can have some adverts. But then selling it to the community and actually having this in depth conversation as to what are the barriers and how we can tackle that*” | Male, young |
|  | “*We can use WhatsApp to share information about meetings… in English but Somali subtitles*” | Male, middle age |
|  | “*It’s better to use media… or somewhere else we could use orally*” | Male, middle age |
| **School & Educational Engagement** | *“The* [school] *nurses, yeah, and then I think because they send out newsletters, but then it’s quite different because not many people will have access to read. So yeah, I think we are good with the verbal stuff, not so much reading”* | Female, young |
|  | “*Even if primary school was a bit more engaged in terms of training parents I think it could be a lot more effective*” | Male, young |
|  | “*The educational curriculum. For example, in Africa what we teach at that age, we teach them in school about diarrhoea and how to manage diarrhoea*” | Male, middle-aged |
| **Group & community Sessions** | “*Sessions for community groups, sessions like here. And maybe not just one time but two year, three year programme*” | Male, middle-age |
|  | “*When we had diabetes we had a lot of community awareness sessions, we also had diabetes experts, we called them champions*” | Male, middle-age |
| **Mother Figure** | “*Without being controversial, the source of that information, within the Somali community, is the mother and I think what is key for us is if the mother is educated enough and knowledgeable enough in vitamin D then the community is educated about it*” | Male, middle-aged |
|  | “*I think that when it goes back to the mum*” | Male, young |
|  | “*Yes, I would say that are the most important person. Yes, they are helpful. Is the mum. The most important person*” | Male, middle-aged |
